# Method-oriented systematic review on the simple scale for acceptance measurement in advanced transport telematics

**DOI:** 10.1371/journal.pone.0248107

**Published:** 2021-03-25

**Authors:** Jan C. Zoellick, Adelheid Kuhlmey, Liane Schenk, Stefan Blüher

**Affiliations:** Charité – Universitätsmedizin Berlin, Corporate Member of Freie Universität Berlin and Humboldt-Universität zu Berlin, Institute of Medical Sociology and Rehabilitation Science, Berlin, Germany; Universitat de Valencia, SPAIN

## Abstract

Acceptance intuitively is a precondition for the adaptation and use of technology. In this systematic review, we examine academic literature on the “simple scale for acceptance measurement” provided by Van der Laan, Heino, and de Waard (1997). This measure is increasingly applied in research on mobility systems without having been thoroughly analysed. This article aims to provide such a critical analysis. We identified 437 unique references in three aggregated databases and included 128 articles (N = 6,058 participants) that empirically applied the scale in this review. The typical study focused on a mobility system using a within-subjects design in a driving simulator in Europe. Based on quality indicators of transparent study aim, group allocation procedure, variable definitions, sample characteristics, (statistical) control of confounders, reproducibility, and reporting of incomplete data and test performance, many of the 128 articles exhibited room for improvements (44% below.50; range 0 to 1). Twenty-eight studies (22%) reported reliability coefficients providing evidence that the scale and its sub-scales produce reliable results (median Cronbach’s α >.83). Missing data from the majority of studies limits this conclusion. Only 2 out of 10 factor analyses replicated the proposed two-dimensional structure questioning the use of these sub-scales. Correlation results provide evidence for convergent validity of acceptance, usefulness, and satisfying with limited confidence, since only 14 studies with a median sample size of *N* = 40 reported correlation coefficients. With these results, the scale might be a valuable addition for technology attitude research. Firstly, we recommend thorough testing for a better understanding of acceptance, usefulness, and satisfying. Secondly, we suggest to report scale results more transparently and rigorously to enable meta-analyses in the future. The study protocol is available at the Open Science Framework (https://osf.io/j782c/).

## Introduction

The “simple scale for acceptance measurement” from colleagues [1] (hereafter referred to as *Simple Scale*) has been widely applied in transportation research. Researchers used the scale as subjective assessments of bicycles [2], helicopters [3], automated driving [4], or various in-car systems ranging from speed adaptation [5] and eco-driving [6] to in-vehicle signs [7] or other driver assistance systems [8]; in online surveys [9], simulators [10], or field trials [11]; in Europe [12, 13], North America [14, 15], or Australia [16]. It was created as “a simple, standard tool for the assessment of acceptance that can be used by the majority of researchers and that allows a comparison of impact of new devices with other systems” [1].

However, aside from the original publication [1], no article systematically investigated the *Simple Scale* regarding reliability, validity, and application contexts. Debates about the *Simple Scale* address the level of data the scale produces ranging from “relative (ordinal) levels of rater acceptance” [17] to Likert-type interval level data [18, 19]. Some authors argue that acceptance includes additional facets other than the dimensions usefulness and satisfying produced by the *Simple Scale* [20]–e.g. perceived ease of use from the technology acceptance model [21] or perceived behavioural control from the theory of planned behaviour [22]. Others [23] see the *Simple Scale* with its limit of being two-dimensional only as a starting point in designing a standardised measure for acceptance. While the scale might be intuitively useful and easy to use, its psychometric characteristics remain unclear.

The purpose of this paper is to understand how the *Simple Scale* is applied, how reliable and valid it is, and what results can be expected when it is used. Those four questions are answered by a method-oriented systematic review on articles that empirically applied the *Simple Scale* in the various contexts listed above. As a result, researchers in transportation science are better informed about the strengths and weaknesses of the *Simple Scale* which improves their work; they can interpret their results before the background of various other applications; and they gain insights into what to expect when they apply the scale. These are the main contributions of this method-oriented systematic review on the *Simple Scale* for acceptance measurement.

### The *Simple Scale*

The original authors define acceptance of a technical system as “direct attitudes towards that system. Attitudes are here defined as predispositions to respond, or tendencies in terms of ‘approach/avoidance’ or ‘favourable/unfavourable’” toward the system [1]. Accordingly, they used nine item pairs spanning a 5-point scale in the format of a semantic differential taken from colleagues’ [24] catalogue of opinion measures (e.g., useful—useless, bad—good, or nice—annoying).

Having tested the measure in six studies and having calculated simultaneous component analyses with varimax rotation between samples (*N* = 291), the authors [1] identified two subscales: usefulness (items 1, 3, 5, 7, and 9) and satisfying (items 2, 4, 6, and 8). They exhibited reliability coefficients (Cronbach’s α) in the range of.73 to.91 for usefulness and.81 to.94 for satisfying. An instruction how best to apply the measure consists of seven steps [1]. The authors suggest (1) an instruction before technology use, (2) an instruction after technology use, (3) coding six items with +2 to -2 and three mirrored items -2 to +2, (4) performing reliability analysis on both sub-scales, (5) calculating means for each item if reliability is sufficient (Cronbach’s α >.65), (6) calculating means for both sub-scales usefulness and satisfying, and (7) calculating difference scores between the pre- and post-measures for both sub-scales [1].

The remainder of this article evaluates the *Simple Scale* and with it the success in developing “a simple, standard tool for the assessment of acceptance that can be used by the majority of researchers and that allows a comparison of impact of new devices with other systems” [1]. Since the *Simple Scale* is increasingly used in recent years [25–28], such a systematic evaluation is necessary to understand its psychometric characteristics and guide authors in further applications. Thus, this paper supports researchers in the field of transportation science interested in subjective evaluations of a system.

### Research questions

We planned and designed the systematic review in accordance with PROSPERO and AMSTAR guidelines for quality enhancement of systematic reviews [29, 30]. It is registered in the Open Science Framework (link: https://osf.io/j782c/). The PRISMA guideline can be found in the S1 File. We did not formulate any restrictions on people, interventions, comparisons, outcomes, and study designs (PICOS). Since this is a method-oriented review, we were primarily interested in the performance of the scale for acceptance measure. In accordance with other method-oriented systematic reviews [31], we formulate the following research questions:

- Q_1_: How do researchers apply the scale?
  - Comparing the contexts and research questions being investigated together with (descriptive or inferential) statistics used to answer them provides insights in the use of this semantic differential.
- Q_2_: How reliable is the scale?
  - Comparing Cronbach’s alphas across studies gives an indication of the scale’s reliability. Additionally, factor extractions and model fit indices in exploratory and confirmatory factor analyses act as parameters to assess whether the scale produces the proposed two-factor structure.
- Q_3_: How valid is the scale?
  - Comparing the studies’ findings regarding correlates provides a measure for discriminant and convergent validity of the scale.
- Q_4_: What are mean results for acceptance measures?
  - Given sufficiently homogeneous scale applications, the weighted average and the distribution of effects give an indication of expected outcomes for the respective application context.

## Methods

### Literature overview

We conducted a systematic literature search on studies empirically applying the *Simple Scale* in May 2018. We searched the following databases:

- EBSCOhost (all databases included),
- Web of Science (Science and Social Science Citation Index), and
- Google Scholar

using the identical search terms:

*A simple procedure for the assessment of acceptance of advanced transport telematics*.

**Fig 1:**
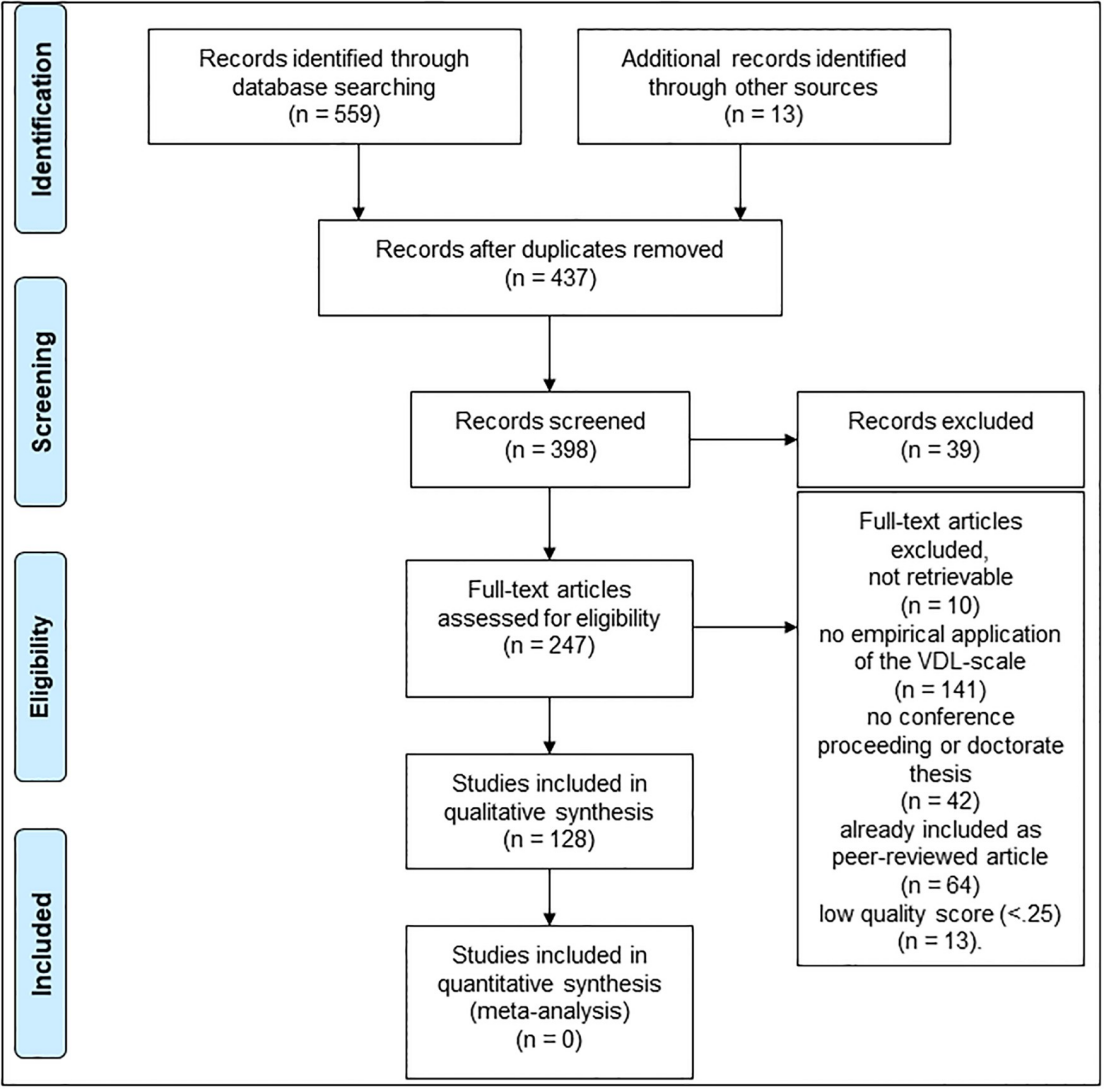
PRISMA diagram of the systematic literature search and exclusion in various points of the process.

In every database, this yielded one search result, namely the original research paper [1]. We marked the option to show all articles that cited this study and exported the resulting lists of citations to a blank Endnote library. With this procedure, we retrieved 559 citations. In successive steps, we reduced this population by removing duplicates, screening the titles and abstracts, and reading their downloaded full texts. All empirical applications of the *Simple Scale* regardless of geographical region were eligible for inclusion (i.e., all translations of the items), as long as the article to be included was written in English or German. We excluded modifications of the scale’s *items*–e.g., replacing “assisting-worthless” with “ugly-attractive” [32] or “nice-annoying” with “not nice-nice” [33]–, but included modifications of the scale’s *range*–e.g., 1 to 5 instead of the original +2 to -2 [34]. In the last step, we screened reference lists of eligible articles to identify further results not listed in the three aggregated databases. We thus added 13 studies to our final population (*N* = 437 without duplicates). Fig 1 presents the PRISMA flow diagram of our literature search. Even with the support of our university librarian, we were unable to retrieve full-texts for ten citations marked in the S2 File. After reading all retrieved full texts, 247 articles remained eligible for inclusion. We included all peer-reviewed articles in the analysis. Additionally, we included all conference proceedings and doctorate theses not already included as journal articles with a quality score ≥.25 (see below). This led to the inclusion of 128 articles for analysis.

### Coding

We coded all 247 empirical applications of the *Simple Scale* according to the first section of our coding manual presented in the S1 Appendix. It provided metadata of the articles including author names, year of publication, title of the study, geographical setting (country of data collection; if not reported, country of first author’s affiliation), institutional link, article type (peer-review journal, conference proceedings, doctorate or graduate theses, reports, and books or book chapters), and journal name in case of peer-review journal publications. We coded the included 128 articles according to the remaining sections of the coding manual. Its second section consisted of the studies’ designs and contents, namely the domain of study, study design (e.g., within- or between-subjects and longitudinal or cross-sectional data collection), research questions, methods, study outcomes, sample size and characteristics (gender and age), and (experimental) conditions. The third section included specifications on the *Simple Scale* applications, namely reports of the scale’s level (e.g., ordinal, interval, or Likert) and range, presentation of scale results (numbers in text or table, bar charts, figures, aggregated or itemwise, or two-dimensional diagram), factor loadings on each subscale, and medians, means, standard deviations, and reliability coefficients of both subscales and the entire scale. The fourth section dealt with relationships of the scale with itself and other constructs and included variables of the analysed model, correlates of the *Simple Scale*, and other statistics. The last section dealt with miscellaneous aspects such as translation and adaptations of the scale, comments, and the team members extracting the data.

The first author coded all included articles. Four team members gave support in coding and discussions. In contrast to other methodological reviews [31], we did not apply independent coding. The resources needed to double-code all 128 articles on 40 categories (at least 5,120 cells on the spreadsheet) would vastly outnumber the benefits of independent coding—particularly since most codes in all sections consisted in copy and paste of the content without assessment and decisions. Merely the code ‘domain of study’ involved category formulation and allocation. This was done in a team meeting with four team members all of which had prior experience in the method.

### Risk of bias quality appraisal

In order to estimate the risk of bias, we assessed the quality of the 128 included articles using eight items from colleagues [35] covering the multiple aspects aim, group allocation procedure, variable definitions, sample characteristics, (statistical) control of confounders, incomplete data, reproducibility, and test performance reporting. Each item provided a score between 0 (criterion not met) and 1 (criterion met). The items and corresponding codes are presented in the S2 Appendix. Each article was independently coded by the first author and one of three other researchers. A set of 30 articles was used as training material. After those were assessed independently, all four coders met to discuss interpretations of the questions and applications of the criteria. After aligning the approaches, the remaining articles were coded independently. This dataset formed the basis for the calculation of Cohen’s kappa as a measure of interrater reliability. Conflicts after completing the quality appraisal were resolved in three meetings between the researchers. We calculated an overall quality score for each article by averaging answers of all applicable items [35]. The overall quality score ranged between 0 (low quality) and 1 (high quality). We used a *t*-test for independent samples to compare quality scores between articles with one group and articles with more than one group (i.e., with between-subjects conditions). We calculated an ANOVA to compare quality scores between the article types “peer-reviewed journal article”, “conference proceeding”, and “doctorate thesis”. For all analyses, we used α = .05 as significance indicator. Lastly, we analysed difficulty (i.e., relative frequency of “criterion met”) and item-scale correlations of the items.

### Statistical analyses

We calculated descriptive statistics of the articles’ metadata, i.e. country and context of origin, or year and type of publication, as well as other features such as sample characteristics, scale range, or presentation of scale results. From these analyses, we could derive typical *Simple Scale* applications suitable to answer our first research question. For participants’ age, we estimated mean ages from categories by assuming equal distribution of individual ages in the categories. Because of incomplete reporting in the study population, we can only partially answer research questions Q_2_-Q_4_ using descriptive analyses and a narrative synthesis instead of planned meta-analytic procedures.

## Results

### Literature review

We identified 437 unique references. Of those, 247 applied the *Simple Scale* empirically– 90 peer-review journal publications, 84 conference proceedings, 32 doctorate or graduate theses, 25 reports, and 16 books or book chapters. An Endnote library with all references can be found in the S2 File next to a spreadsheet with codes for section A of the coding manual for 247 articles (S1 Dataset). We included peer-reviewed journal articles, conference proceedings, and doctorate theses (*N* = 128) in further analyses.

The combined sample size of the 128 studies was *N* = 6,058 (range 3 to 387; median 32). Note that in some cases the same dataset had been used for more than one publication (e.g., *N* = 72 in [36 and 37]), and that some articles theses used more than one sample in more than one study (e.g., [38–42]). Of all studies reporting gender distribution (112 articles; *N* = 5,462), 57% of participants were male. Mean age of participants was *M* = 37.15 years in 100 studies (*N* = 4,289 participants) reporting means. Estimated mean age for participants was *M* = 37.62 years in 14 studies (*N* = 546 participants) only reporting age categories. The remaining studies with *N* = 1,223 participants did not report age in a way to estimate a mean.

### Quality appraisal

Cohen’s kappas between the first author and the other three researchers were.53,.54, and.71 before, and 1, 1, and 1 after conflict resolution, respectively. The largest discrepancies in appraisal were in item 3, item 5, and item 7 with 59%, 61%, and 71% initial agreement, respectively. Item statistics are displayed in Table 1. Codes for each article and item can be found in the S2 Dataset. The quality appraisal tool had a reliability of Cronbach’s α = .47 suggesting that these items do not form a narrow, one-dimensional construct of quality. This is exactly as expected since we aimed to include different facets of quality not contingent on another.

**Table 1:** Item statistics of the eight-item quality appraisal tool.

#	Item	N	Mean	SD	Difficulty^1^	Item-scale correlation
1	Aims of the study stated	128	.90	.30	.90	.19^a^ (.05^b^)
2	Group allocation reported	58	.67	.47	.67	.27^a^
3	Major variables defined	128	.25	.36	.13	.14^a^ (.30^b^)
4	Study characteristics described	128	.82	.31	.71	.21^a^ (.16^b^)
5	Participant characteristics controlled	122	.29	.39	.18	.41^a^ (.11^b^)
6	Protocol violations and missing data reported	126	.39	.47	.35	.10^a^ (.19^b^)
7	Procedure clear to be reproduced	128	.91	.20	.84	.24^a^ (.19^b^)
8	Test performance reported	128	.26	.44	.26	.19^a^ (.16^b^)

^1^ relative frequency of “criterion met”.

^a^ Item-scale correlation for all eight items for studies with ≥ 2 groups (*N* = 56 studies).

^b^ Item-scale correlation with seven items excluding item 2 (N = 121 studies).

Overall, quality scores were low (*M* = .55, *SD* = .17; scale range 0 to 1). Sixty-five articles (51%) retrieved a score above.50. The majority of studies reported their aims (item 1) and procedure to be reproduced (item 7) at least partially. Surprisingly, a minority of studies defined the constructs they used to fulfil their stated aim (item 3) and reported test performance indicators such as Cronbach’s α (item 8). Low quality scores mean that the study is more difficult to interpret and reproduce, because important information is missing.

Quality scores among articles with at least two groups based on between-subjects conditions (58 studies) did not differ from those among articles with only one group without between-subjects conditions (70 studies) (*t*(126) = 1.27, *p* = .207). Quality scores were highest for doctorate theses (*M* = .60, *SD* = .16; 10 studies) followed by peer-reviewed journal articles (*M* = .56, *SD* = .18; 90 studies) and conference proceedings (*M* = .51, *SD* = .15; 28 studies), however without significant differences (*F*(2,125) = 1.56; *p* = .214).

Coding of item 6 was difficult since an absence of protocol violation and missing data *documentation* might also be the result of no protocol violation and no missing data. However, this would mean that the majority of studies had no missing data whatsoever—a far-fetched assumption for empirical attitude research. Removing item 6 from the overall quality score calculation resulted in slightly improved quality scores across articles (*M* = .58, *SD* = .17) with 69 articles (54%) receiving a quality score above.50.

### Applications of the *Simple Scale* (Q_1_)

Our first research question addressed the application of the scale regarding the studies’ meta-data. The 128 articles were published by 313 different authors. For peer-reviewed journal articles, journals with the most publications were *Transportation Research*: *Part F* (23), *Accident Analysis & Prevention* (14), *Applied Ergonomics* (7), and *Human Factors* (6). The 128 articles spanned 22 years of research with a focus on the recent years (57% of articles published since 2014). Most applications of the scale emerged from technical and engineering departments of research institutions focusing on transportation. We identified 17 topics of focus in the included articles with driver assistance systems (45), automated driving (21), intelligent speed adaptation (14), vehicle safety systems (11), and electric vehicles (11) being the most frequent.

Geographically, the 128 studies were conducted in 15 different countries with 75% of studies emerging from Germany (40), The Netherlands (30), USA (18), and the UK (14). Most studies collected data within subjects (80), some between subjects (15), and the remaining studies within and between subjects (38). Seven studies additionally used a longitudinal design over multiple weeks or months. The vast majority of studies used a (driving) simulator (77), field trials (39), or both (3). The remaining studies relied on online questionnaires or in-lab mock-up equipment other than simulators.

Most publications did not test theoretical models with variables explaining certain outcomes such as system use or acceptance [43, 44]. Instead, the typical application of the *Simple Scale* consisted in its loose connection with a paper otherwise concerned with technical aspects of a new system in transportation. Here, speed, lateral offset, absolute driver torque, steering wheel angle, glace duration, or reaction times were assessed to evaluate the system’s performance. It seemed the *Simple Scale* was an add-on to enhance technical arguments with a subjective assessment from the users. This is exemplified by colleagues [8] who after explicating technical aspects and tests at length stated “[i]n addition, subjective evaluations were conducted to check for system acceptance”. Articles centring on acceptance such as [9] (“[t]he core of this work is an extensive SEM analysis on the factors driving smart charging acceptance”) were the exception.

Consequently, application, reporting, and presentation of the scale’s results varied and were in many cases incomplete. Seventeen studies used a different scale range than the original -2 to 2 (e.g., 1 to 5, 1 to 7 or -50 to 50), and 18 studies did not report the scaling leaving 93 studies (73%) reportedly using the *Simple Scale* in its original scale range. Twenty-one studies erroneously reported that the semantic differential consisted of Likert-scales. Some studies adopted the items to form an actual Likert-scale measuring (dis-)agreement [34].

Only eight studies reported factor analyses to test the two-dimensional structure of the *Simple Scale*, and only 28 studies reported reliability coefficients as a measure for scale accuracy (see next section). Nonetheless, 78 studies formed means corresponding to the two sub-scales usefulness and satisfying *without reporting* whether data structure and scale characteristics allow for this procedure. Six studies reported the scale’s or sub-scales’ medians.

The majority of studies (73) reported descriptive statistics of the *Simple Scale* as numbers in tables or text. The remaining studies used illustrations such as a two-dimensional diagram with the two sub-scales usefulness and satisfying as dimensions (21), bar charts (18), or other figures (11). Three studies used plain text, and the remaining three studies did not report descriptive statistics from the *Simple Scale*.

Fourteen studies reported relationships between the *Simple Scale* and other constructs resulting in 70 estimates. We used these for answering the third research question below. Table 2 presents all 128 articles with the information listed above. A spreadsheet with codes for all 128 articles can be found in the S3 Dataset.

**Table 2:** Metadata, quality scores, sample characteristics, and *Simple Scale* application of 128 articles.

Authors	Country	Domain^1^	Design^2^^/^^3^	Quality	*N*	Gender (% men)	Age M (SD)	Depicting results^4^	Scale range	Correlates of *Simple Scale*
Adell, Várhelyi, Alonso, et al. (2008) [45]	ESP, SWE	DAS	3 / 4	.25	123	N/A	N/A	3	-50 to 50	N/A
Adell et al. (2011) [46]	ITA	DAS	1 / 2	.64	19	53%	45.47^d^	1	-50 to 50	N/A
Adell, Várhelyi, della Fontana, et al. (2008) [47]	FRA	DAS	3 / 1	.36	34	88%	N/A	3	-5 to 5	N/A
Adell, Várhelyi & Hjälmdahl (2008) [48]	HUN, ESP	ISA	3 / 2	.56	37	81%	43.03	1	N/A	N/A
Albert et al. (2015) [49]	GER	AUT	1 / 2	.64	37	84%	32.47 (5.41)	3	-2 to 2	N/A
Beggiato & Krems (2013) [13]	GER	DAS	1^+^ / 1	.88	51	49%	24.00 (2.37)	4	-2 to 2	N/A
Beggiato et al. (2015) [50]	GER	DAS	1^+^ / 2	.71	15	53%	28.00 (1.82)	4	-2 to 2	N/A
Bellotti et al. (2005) [51]	ITA, SWE	HMIF	1 / 12	.29	32	50%	32.00	5	N/A	N/A
Black et al. (2018) [25]	GER^a^	ORI	2 / 4	.25	26	69%	29.00	1	-2 to 2	N/A
Blömacher et al. (2018) [52]	GER^a^	AUT	1 / 4	.63	120	43%	39.40 (11.78)	1	-2 to 2	N/A
Brookhuis & de Waard (1999) [53]	NED	ISA	3 / 1	.38	24	N/A	N/A	1	-2 to 2	N/A
Brookhuis & Dicke (2009) [12]	NED	DAS	3 / 1	.50	24	67%	29.65	2	-2 to 2	N/A
Brookhuis et al. (2009) [19]	NED	DAS	1 / 1	.50	37	81%	41.50^d^	2	-2 to 2	N/A
Breugelmans et al. (2009) [54]	NED^a^	DAS	1 / 1	.44	18	N/A	25,90 (5.40)	1	-2 to 2	N/A
Bueno et al. (2014) [18]	FRA^a^	VSS	2 / 1	.81	36	50%	31.10	1	-2 to 2	N/A
Bühler, Cocron et al. (2014) [55]	GER	EV	1^+^ / 2	.86	79^b^ ^c^	85%	49.00 (9.60)	1	-2 to 2	Use × Sat:.55 ≤ *r* ≤.82
										Use × Gen. perception.:.47 ≤ *r* ≤.61
										Sat × Gen. perception:.43 ≤ *r* ≤.61
Bühler, Franke et al. (2014) [56]	GER	EV	1^+^ / 2	1.00	35	11%	47.30	4	-2 to 2	N/A
Chen et al. (2007) [57]	SWE	DAS	1 / 1	.57	7	100%	N/A	3	-2 to 2	N/A
Cocron et al. (2013) [58]	GER	EV	1^+^ / 2	.71	40^b^ ^c^	88%	50.00 (10.20)	1	-2 to 2	Preliminary analyses of Bühler et al. (2014)
Cocron et al. (2011) [59]	GER	EV	1^+^ / 2	.25	39^b^ ^c^	82%	48.63 (8.76)	1	-2 to 2	Preliminary analyses of Bühler et al. (2014)
Comte (2000) [60]	UK	ISA	2 / 1	.25	40	50%	34.00	2	N/A	N/A
Comte & Jamson, A.H. (2000) [61]	UK	ISA	1 / 1	.21	30	50%	31.00	2	-2 to 2	N/A
Comte et al. (2000) [62]	UK	ISA	1 / 2	.29	8	N/A	N/A	2	N/A	N/A
Cottrell & Barton (2012) [11]	USA	DSTR	1 / 2	.71	72	42%	24.04	1	N/A	Acc × age: -.16 ≤ *r* ≤.15
										Acc × years licensed: -.14 ≤ *r* ≤.15
Creaser & Manser (2013) [7]	USA	IVS	2 / 1	.63	60	55%	44.35 (15.31^e^)	3	-2 to 2	N/A
Creaser et al. (2007) [63]	USA	VSS	3 / 1	.63	48	60%	44.23 (18.89^e^)	3	-2 to 2	N/A
Davidse et al. (2009) [64]	NED	DAS	3 / 1	.63	40	75%	48.38 (23.07^e^)	5	N/A	N/A
de Boer et al. (2010) [65]	NED	VSS	1 / 3	.57	88	60%	31.18	2	-2 to 2	N/A
de Waard & Brookhuis (1997) [66]	NED	VSS	2 / 2	.50	22	79%	38.00 (7.10)	2	-2 to 2	N/A
de Waard et al. (2004) [67]	NED	AUT	3 / 2	.38	25	N/A	N/A	4	-2 to 2	Use × trust: *r* = .43
de Waard et al. (2009) [68]	NED	DAS	1 / 1	.69	33	82%	49.28 (21.24^e^)	1	-2 to 2	N/A
de Waard, van der Hulst & Brookhuis (1999) [69]	NED	VSS	2 / 2	.75	27	78%	46.67 (14.55^e^)	2	-2 to 2	N/A
de Waard, van der Hulst, Hoedemaker et al. (1999) [70]	NED	AUT	1 / 1	.43	20	80%	29.80 (6.00)	1	N/A	N/A
Dijksterhuis et al. (2012) [71]	NED	DAS	1 / 1	.64	31	84%	26.10 (4.40)	3	-2 to 2	N/A
Donmez et al. (2007) [72]	USA	VSS	3 / 1	.75	29	48%	30.30 (12.73^e^)	3	-2 to 2	N/A
Donmez et al. (2008) [73]	USA	VSS	3 / 1	.38	48	52%	20.25 (0.81^e^)	3	-2 to 2	N/A
Donmez et al. (2006) [74]	USA	DDTR	3 / 1	.50	28	N/A	55.29 (^c^)	3	-2 to 2	Use × trust: *ρ* = .73
										Sat × trust: *ρ* = .63
Dotzauer et al. (2013) [75]	NED	DAS	3^+^ / 1	.69	18	N/A	72.60 (3.70^e^)	1	-2 to 2	N/A
Drew & Hayes (2012) [76]	USA	DAS	1 / 1	.50	24	50%	38.00 (18.00)	1	-2 to 2	N/A
Drucker (2013) [77]	USA	DAS	3 / 1	.56	85	49%	45.44	3	-2 to 2	N/A
Dubbeldam et al. (2017) [2]	NED	CYC	1 / 2	.43	9	56%	74.00 (5.00)	1	-18 to 18	N/A
Duffield & Krupenia (2015) [78]	SWE	DAS	1 / 1	.43	15	87%	41.00	3	-2 to 2	N/A
Engelbrektsson & Karlsson (2012) [79]	SWE	DAS	1 / 2	.50	96	60%	N/A	4	-2 to 2	N/A
Eriksson, Banks et al. (2017) [80]	UK	AUT	2 / 12	.50	38	58%	30.92 (9.26^e^)	1	N/A	Use × use (two samples): *r* = -.15
										Sat × sat (two samples): *r* = -.25
Eriksson, Petermeijer, et al. (2017) [81]	UK	AUT	1 / 1	.64	25	56%	25.70 (3.90)	1	-2 to 2	N/A
Fagerlönn et al. (2017) [82]	SWE	HMIF	1 / 2	.50	17	82%	47.00 (11.00)	1	-2 to 2	N/A
Fischer et al. (2014) [83]	GER	HMIA	2 / 1	.38	56	84%	28.54	1	-2 to 2	N/A
Franke et al. (2017) [37]	GER	EV	1 / 2	.79	72^c^	83%	42.80 (9.50)	6	N/A	N/A
Franke et al. (2016) [36]	GER	EV	1 / 2	.86	72^c^	83%	42.80 (9.50)	1	N/A	N/A
Gauerhof et al. (2015) [84]	GER	DAS	1 / 4	.50	21	N/A	25.40 (1.79)	1	-2 to 2	N/A
Giang et al. (2014) [85]	CAN	HMIA	1 / 1	.57	6	50%	25.50 (3.33)	6	N/A	N/A
Günther et al. (2016) [86]	GER	EV	3 / 2	.75	61	79%	37.13 (9.67)	2	-2 to 2	N/A
Hartwich et al. (2018) [87]	GER	AUT	3 / 1	.88	40	53%	48.50 (21.23^e^)	2	-2 to 2	Acc × comfort: *r* = .71
										Acc × enjoyment: *r* = .38
Hegeman et al. (2007) [88]	NED	DAS	1 / 1	.71	24	50%	45.00 (9.00)	1	-2 to 2	N/A
Heinig (2009) [89]	GER	ISA	3 / 2	.88	36	53%	28.30	1	-2 to 2	N/A
Henzler et al. (2015) [90]	GER	DAS	1 / 2	.71	24	96%	43.60 (9.40)	1	N/A	N/A
Heyes et al. (2015) [91]	GER	ECO	3 / 2	.38	40	N/A	N/A	1	-2 to 2	N/A
Hibberd et al. (2015) [6]	UK	ECO	1 / 1	.50	24	50%	37.15 (14.34^e^)	2	-2 to 2	N/A
Hjälmdahl et al. (2017) [92]	SWE	AUT	1 / 1	.36	24	100%	N/A	3	-2 to 2	Use × QUIS: *r* = .80
										Sat × QUIS: *r* = -.89
Hock et al. (2016) [93]	GER	AUT	2 / 1	.63	38	39%	24.00 (3.54)	1	1 to 7	N/A
Hoedemaeker & Brookhuis (1998) [94]	NED	DAS	1 / 1	.50	38	66%	42.50^d^	1	-2 to 2	N/A
Houtenbos et al. (2017) [26]	NED	DAS	1 / 1	.64	25	72%	51.10 (12.90)	4	1 to 5	N/A
Jagiełłowicz-Kaufmann (2016) [95]	GER	EV	1 / 1	.43	19	N/A	N/A	3	-3 to 3	N/A
Jamson, A.H. et al. (2008) [96]	UK	VSS	3 / 1	.63	45	51%	37.40 (13.90)	5	-2 to 2	N/A
Jamson, S.L. (2006) [97]	UK	ISA	3 / 12	.50	18	50%	27.89	1	N/A	Acc × DSQ: *r* = -.46 & *r* = -.30
										Acc × Speed: *r* = -.56 & *r* = -.44
										Acc × Usage: *r* = .23 & *r* = .52
										Acc × Age: *r* = .12 & *r* = .22
Jamson, S.L. et al. (2012) [5]	UK	ISA	1 / 1	.36	26	46%	40.31^d^	2	-2 to 2	N/A
Jiménez et al. (2016) [98]	ESP	VSS	1 / 1	.50	20	50%	35.35 (13.23)	2	N/A	N/A
Jizba, T. (2017) [99]	CZE	DAS	1 / 1	.43	18	67%	32.50 (10.05)	1	-2 to 2	N/A
Kidd (2012) [100]	USA	VSS	3 / 1	.81	80	50%	40.65 (13.44^e^)	1	-2 to 2	N/A
Köhler et al. (2014) [101]	GER	DSTR	1 / 2	.57	27	59%	35.93 (12.70)	1	-2 to 2	N/A
Körber et al. (2018) [27]	GER	AUT	3 / 1	.88	40	50%	25.20 (2.60)	1	-2 to 2	N/A
Kotte et al. (2016) [102]	GER	EV	1 / 1	.43	27	70%	35.37^d^	3	-2 to 2	N/A
Koustanaï et al. (2012) [103]	FRA	DAS	2 / 1	.69	28	71%	41.25 (9.83^e^)	1	N/A	N/A
Krahnstöver (2017) study 1 [40]	GER	DAS	1 / 1	.71	76	74%	39.60 (10.90)	1	-2 to 2	N/A
Krahnstöver (2017) study 2 [40, 104]	GER	DAS	1 / 2	.71	39	62%	42.20 (12.60)	1	-2 to 2	N/A
Langer et al. (2016) [105]	GER	DAS	1 / 2	.64	21	57%	31.60 (3.50)	1	-2 to 2	N/A
Liao (2013) [15]	USA	EVHS	1 / 2	.50	18	61%	44.20 (15.20)	1	-2 to 2	N/A
Ma & Zhou (2016) [106]	USA	IVS	1 / 1	.42	21	62%	33.89^d^	1	-2 to 2	N/A
Madigan et al. (2018) [28]	UK	AUT	1 / 1	.57	29	52%	34.21 (8.94)	2	N/A	N/A
McIlroy, Stanton & Godwin (2017) [107]	UK	ECO	1 / 1	.64	24	58%	34.71 (13.08)	2	-2 to 2	N/A
McIlroy, Stanton, Godwin et al. (2017) [108]	UK	ECO	1 / 1	.50	30	57%	33.83 (11.95)	2	-2 to 2	N/A
Melman et al. (2017) [109]	NED	DAS	1 / 1	.43	24	71%	28.00 (9.60)	1	-2 to 2	Use × Sat:.54 ≤ *ρ* ≤.70
										Use × Workload: -.50 ≤ *ρ* ≤ -.24
										Sat × Workload: -.62 ≤ *ρ* ≤ -.25
Merrikhpour (2017) study 1 [38]	CAN	DDTR	3 / 1	.69	29	52%	18,21 (0.53)	1	-2 to 2	N/A
Merrikhpour (2017) study 2 [38]	CAN	DDTR	3 / 1	.69	40	48%	18.38 (0.29)	1	-2 to 2	N/A
Müllhäuser (2018) [3]	GER	HELI	1 / 4	.33	3	N/A	N/A	3	-1 to 1	N/A
Nordhoff et al. (2018) [4]	GER	AUT	1 / 2	.86	274	62%	34.90 (14.20)	4	1 to 5	N/A
Othersen, I. (2016) [110]	GER	AUT	3 / 2	.69	24	75%	36.50 (9.80)	1	0 to 5	N/A
Perelló et al. (2016) [111]	Spain	ECO	1 / 1	.50	30	87%	33.67 (5.55)	1	N/A	N/A
Petermeijer et al. (2015) [10]	NED	DAS	1 / 1	.57	32	81%	25.80 (3.30)	1	-2 to 2	N/A
Petermeijer, Bazilinskyy et al. (2017) [112]	GER	AUT	1 / 1	.71	24	67%	27.90 (3.00)	1	-2 to 2	N/A
Petermeijer, Cieler et al. (2017) [113]	GER	AUT	1 / 1	.71	18	72%	43.00 (15.20)	4	-2 to 2	N/A
Petermeijer, Doubek et al. (2017) [114]	GER	AUT	2 /	.38	101	69%	24.68	1	-2 to 2	N/A
Pinotti et al. (2014) [115]	ITA^a^	HMIF	1 / 1	.50	59	61%	37.80 (12.60)	1	-2 to 2	N/A
Politis et al. (2018) [116]	UK^a^	HMIF	1 / 1	.36	49	51%	45.51 (17.36)	1	-2 to 2	N/A
Prasch & Tretter (2016) [117]	GER	AUT	1 / 3	.36	36	56%	25.60 (6.30)	2	-2 to 2	N/A
Rahman et al. (2017) [20]	USA	DAS	1 / 3	1.00	387	52%	35.60 (11.00)	1	N/A	Acc × Behav. Intent.: *r* = .89
										Acc × PU: *r* = .88
										Acc × PEoU: *r* = .49
										Acc × Social norms: *r* = .58
										Acc × Behav. control: *r* = .45
										Acc × Perform. expectancy: *r* = .86
										Acc × Effort expectancy: *r* = .46
Rakauskas et al. (2010) [118]	USA	DAS	1 / 4	.36	13	46%	28.50 (9.80)	3	-2 to 2	N/A
Rakauskas et al. (2005) [119]	USA	DAS	1 / 2	.43	9	89%	39.00	4	-2 to 2	N/A
Risto (2014) study 1 [39]	NED	TFA	1 / 3	.71	237	76%	N/A	1	-2 to 2	N/A
Risto (2014) study 2 [39]	NED	TFA	1 / 1	.71	35	86%	46.90 (12.10)	2	-2 to 2	N/A
Rook & Hogema (2005) [120]	NED	ISA	2 / 1	.38	64	91%	39.95 (8.51^e^)	1	-2 to 2	N/A
Saito et al. (2016) [121]	JAP	DAS	1 / 1	.43	15	53%	68.50 (3.07)	1	-2 to 2	N/A
Sasangohar et al. (2015) [14]	CAN	CARE	1 / 2	.64	28	N/A	N/A	1	-2 to 2	N/A
Sayer et al. (2007) [122]	USA	DAS	1 / 2	.29	78	50%	44.50^d^	1	-2 to 2	Use × Sat:.72 ≤ *r* ≤.81
Schieben et al. (2014) [123]	GER	DAS	2 / 1	.75	40	68%	37.53^d^ (12.21^e^)	1	-2 to 2	N/A
Schmalfuß et al. (2017) [42]	GER	EV	3 / 23	.88	316^b^	45%	27.60 (11.30)	1	-2 to 2	Use × Willing. to purchase: *r* = -.33
										Sat × Willing. to purchase: *r* = .15
Shahab (2014) study 1 [41]	NED	DAS	1 / 1	.56	29	76%	29.00^d^	1	-2 to 2	N/A
Shahab (2014) study 2 [41]	NED	DAS	2 / 4	.56	28	78%	23.25	2	-2 to 2	N/A
Shen & Neyens (2017) [34]	USA	DAS	3 / 1	.50	48^b^	50%	21.17 (1.91)	1	1 to 5	N/A
Shyrokau et al. (2018) [124]	NED	HMIA	3 / 1	.56	32	100%	34.41 (14.99^e^)	1	-2 to 2	N/A
Simon et al. (2015) [125]	GER	AUT	1 / 3	.69	83	61%	32.20(10.90)	1	1 to 5	Acc × age: *r* = neg
										Acc × years of driver license: *r* = neg Acc × km driven annually: *r* = neg
Spyropoulou et al. (2014) [126]	UK	ISA	1 / 1	.43	23	78%	33.20 (8.57)	1	-2 to 2	N/A
Stahl et al. (2016) [127]	CAN	VSS	3 / 1	.69	48	N/A	29.85 (14.52^e^)	1	-2 to 2	N/A
Staubach et al. (2014) [128]	GER	ECO	1 / 1	.57	30	50%	41.50 (15.20)	1	-2 to 2	N/A
Tijerina et al. (2016) [129]	USA^a^	AUT	3 / 1	.38	34	68%	N/A	1	-2 to 2	N/A
Urhahne (2016) [130]	GER	DAS	1 / 2	.44	10	70%	44.50^d^	1	-2 to 2	N/A
van den Beukel & van der Voort (2017) [131]	NED	AUT	1 / 1	.50	24	N/A	26.70	1	1 to 5	N/A
van den Beukel et al. (2016) [132]	NED	AUT	1 / 1	.50	37	54%	47.00 (12.60)	1	1 to 7	N/A
van Driel et al. (2007) [133]	NED	DAS	3 / 1	.36	37	N/A	43.00 (10.00)	2	-2 to 2	N/A
van Nes et al. (2010) [134]	NED	DAS	3 / 1	.69	46	67%	37.67^d^	4	-2 to 2	N/A
Van Oosterhout et al. (2018) [135]	NED	HMIA	3 / 4	.56	36	100%	30.00^d^	3	-2 to 2	N/A
Várhelyi et al. (2015) [136]	ITA	DAS	1 / 2	.43	24	54%	41.17^d^	3	-50 to 50	N/A
Verberne et al. (2012) [137]	NED	DAS	3 / 4	.56	61	67%	N/A	1	-2 to 2	N/A
Vlassenroot et al. (2007) [138]	BEL	ISA	3 / 2	.44	62^b^	68%	N/A	3	N/A	N/A
Vlassenroot et al. (2010) [139]	BEL, NED	ISA	1 / 3	.71	148	N/A	N/A	1	N/A	Use × Acceptability: -.53 ≤ *r* ≤.69
										Sat × Acceptability: ±.54 ≤ *r* ≤.69
										Use × ISA-effect: *r* = -.26 & *r* = ±.40
										Sat × ISA-effect: *r* = -.28 & *r* = .42
										Use × ITS-useful: *r* = -.26 & *r* = .44
										Sat × ITS-useful: *r* = -.46 & *r* = -.30
										Use × Sat: ±.96 ≤ *r* ≤.93
										*Note*: inconsistent signs (±)
Wang et al. (2016) [140]	CHN, SWE	DAS	3 / 1	.38	45	56%	34.78^d^	1	-2 to 2	N/A
Wang et al. (2017) [141]	SWE	DAS	1 / 1	.43	30	60%	40.00	1	-2 to 2	N/A
Will & Schuller (2016) [9]	GER	EV	1 / 3	.71	237	90%	N/A	4	1 to 5	N/A
Winkler et al. (2018) [8]	GER	DAS	3 / 1	.50	24	46%	26.80 (8.20)	1	-2 to 2	N/A
Wolter (2017) [142]	GER	DAS	1 / 2		29	83%	38.00	1	-2 to 2	N/A
Young et al. (2010) [16]	AUS	ISA	3 / 1	.56	30	57%	29.35 (10.27^e^)	3	-2 to 2	N/A
Zhao & Wu (2013) [143]	ESP, SWE	ISA	3 / 1	.38	40	50%	31.80 (5.04)	1	-2 to 2	N/A
*Total*				*M* = .55	6,058	58%	37.15 (N = 4,289)			

N/A, not reported.

^1^ DAS, driver assistance system; AUT, automated driving; ISA, intelligent speed adaptation; VSS, vehicle safety systems; EV, electric vehicles; ECO, eco-driving; CARE, care and nursing; CYC, cycling; DSTR, driver stress; DDTR, driver distraction; HELI, helicopters; EVHS, extra-vehicular human safety; HMIF, human-machine interface; HMIA, human-machine interaction; IVS, in-vehicle signs; ORI, operating room interactions; TFA, traffic flow assistant.

^2^ 1, within subjects; 2, between subjects; 3, within and between subjects; ^+^, longitudinal.

^3^ 1, (driving) simulator; 2, field trial; 3, online survey; 4, lab-test with mock-up equipment.

^4^ 1, numbers; 2, bar chart; 3, two-dimensional diagram; 4, figure; 5, plain text; 6, no reporting.

^a^ Country retrieved by first author affiliation.

^b^ Inconsistencies in sample size reporting or only sub-sample analysed.

^c^ Same sample in multiple publications.

^d^ Mean age estimate calculated from age categories using the formula $\frac{1}{N}\sum \left(\frac{\left({min}_{i}+{max}_{i}\right)}{2}*n_{i}\right)$.

^e^ Standard deviation estimate calculated from categories using the formula $\sqrt{\frac{\sum \left((n_{i}-1)*{SD}_{i}^{2}+n_{i}*({\overset{-}{x}}_{i}-{\overset{-}{X})}^{2}\right)}{N-1}}$.

^f^ The reported *SD* = 11.3 for the age group 65–75 years lies outside the mathematically possible range; thus we refrained from estimating total SD.

### Reliability (Q_2_)

The second research question addressed the reliability of the scale. The original authors [1] argued that Cronbach’s α >.65 suffices for the sub-scales’ reliabilities. However, recent articles argue for *increased* lower *and* upper limits of reliability whilst criticising Cronbach’s α as a measure that overestimates reliability if its assumptions are violated [144–146]. Based on these debates, we consider values of Cronbach’s α ≥.80 as acceptable measures for reliability of established scales.

**Fig 2:**
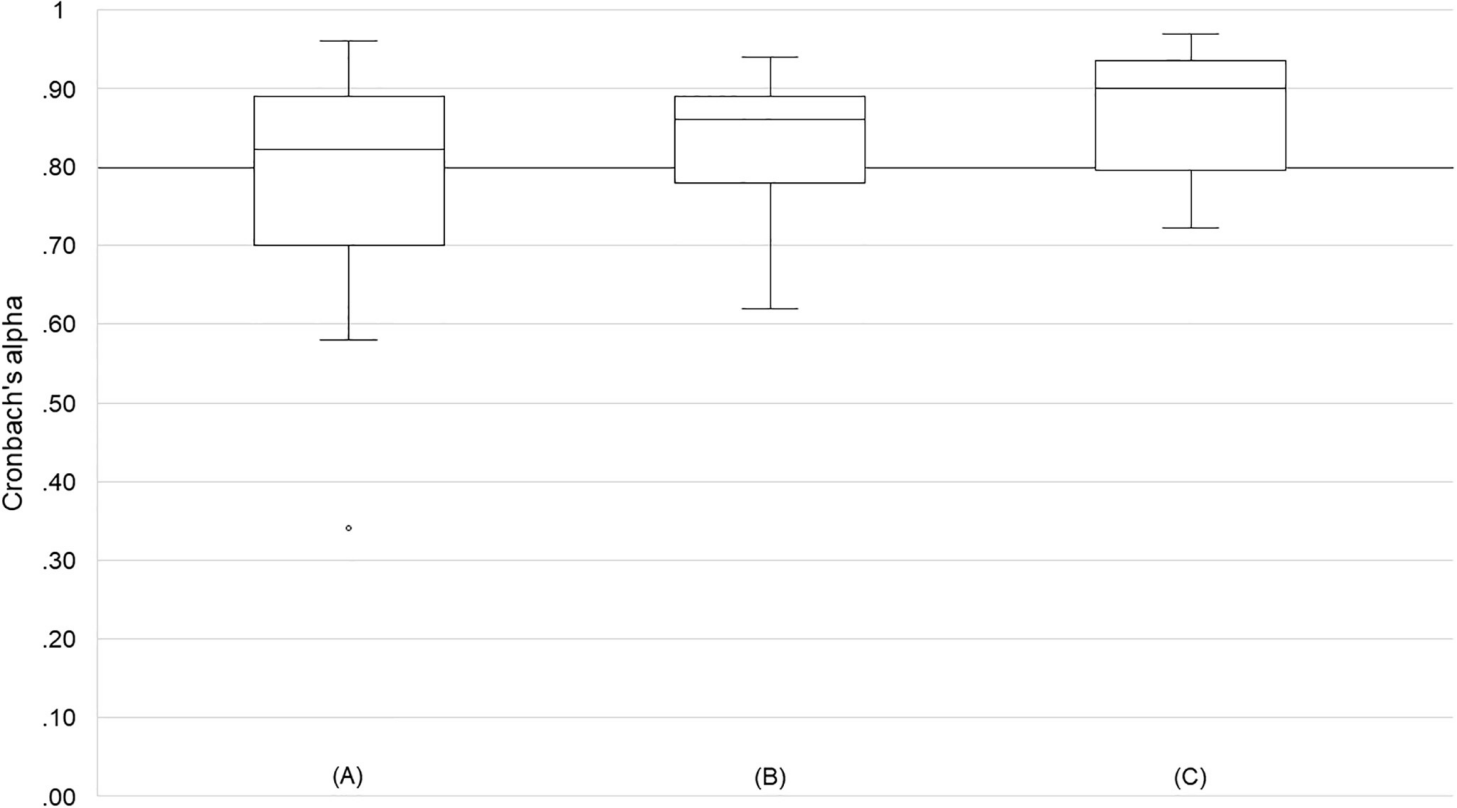
Boxplots of Cronbach’s α for the two sub-scales and the entire scale. (A) usefulness, (B) satisfying, (C) entire scale. The threshold of Cronbach’s α = .80 is highlighted.

Reporting of reliability among the included articles was sparse. Only 65 Cronbach’s α coefficients were reported in 21 studies (Table 3) [11, 14, 20, 27, 36, 37, 40, 42, 52, 57, 61, 62, 69, 89–91, 106, 122, 126, 135, 137]. Seven additional studies calculated multiple Cronbach’s α coefficients for different study conditions, but reported only the upper and/or lower limits [13, 38, 50, 55, 56, 87, 134]. Box plots for the distribution of Cronbach’s α coefficients for both sub-scales and the entire scale are depicted in Fig 2. We included the upper and lower limits of the reported ranges as two Cronbach’s α coefficients for calculating descriptive statistics and modelling the box plots.

**Table 3:** Reliability coefficients of the *Simple Scale* and its two sub-scales usefulness and satisfying across 28 studies.

Concepts	*N* studies	*N* sample	*N* coefficients	Median (α)	Mean (α)	SD (α)	Range (α)
Usefulness	18	1,110	35	.82	.79^a^	.13^a^	.34 -.93
					.91^b^		
Satisfying	18	1,110	35	.86	.83^a^	.08^a^	.62 -.94
					.84^b^		
Acceptance	10	712	13	.90	.87^a^	.07	.72 -.94
					.91^b^		

The studies reporting coefficients for the sub-scale usefulness were identical with those reporting coefficients for the sub-scale satisfying.

α = Cronbach’s alpha.

^a^ unweighted.

^b^ weighted with sample size.

Based on the median coefficients and the weighed means, the *Simple Scale* and its two sub-scales usefulness and satisfying can be seen as reliable. However, missing data from 100 studies limits the certainty of these results considerably.

Only eight studies with *N* = 869 participants calculated a total of ten explorative factor and principal component analyses. Two of these analyses yielded the aspired two factors usefulness and satisfying [9, 13]. Three factor analyses resulted in only one factor named acceptance [36, 37, 50, 87]. In one article [139], four factor analyses were calculated. Three times all items from the usefulness sub-scale formed a factor, and once all items from the satisfying sub-scale loaded on one factor. Another article [4] used principal component extraction on 67 items (including the *Simple Scale*) that produced the components *intention to use*, *shuttle and service characteristics*, and *shuttle effectiveness*. Items 1, 3, 5, and 8 of the *Simple Scale* loaded on the component intention to use; all other items did not load on any of the three components.

These results stand in contrast to the original authors’ [1] own simultaneous component analyses across six samples (*N* = 291), which demonstrated the two-dimensional scale structure. This result cannot be replicated easily in other applications of the scale. No study reported confirmatory factor analyses or model fit indices.

### Validity (Q_3_)

The third research question addressed validity via correlations with closely related constructs. Fourteen studies (*N* = 1,360) reported correlations of the *Simple Scale* and its two sub-scales with other constructs. Together, they reported 70 correlation coefficients with 22 other constructs. Unfortunately, there was almost no overlap in correlations between studies.

The two sub-scales were correlated in four studies providing coefficients of *r* = .55, *r* = .62, and *r* = 82 [55], *ρ* = .54, *ρ* = .63, and *ρ* = .70 [109], *r* = .72, *r* = 75, and *r* = 81 [122], and *r* = ±.96, *r* = ±.93, and *r* = .93 [139]. These last coefficients were surprisingly high and reported with conflicting signs limiting trust in these estimates. Nonetheless, results indicate that usefulness and satisfying are closely related concepts.

The entire scale correlated with other measures of usefulness, e.g. perceived usefulness from TAM *r* = .88 and performance expectancy from UTAUT *r* = .86 [20], as well as with other measures of satisfaction, e.g. comfort *r* = .71 and enjoyment *r* = .38 [87]. These results indicate convergent validity of the *Simple Scale* [147].

A limitation of these correlations were sample sizes of median *N* = 40 in the 14 studies. Such a low *N* reduces test power considerably. We refer to colleagues who have demonstrated the effect of small sample sizes on the informative value of correlation analyses [148, 149].

### Acceptance scores (Q_4_)

The fourth research question addressed the values of the *Simple Scale* across studies within homogeneous application scenarios. In total, 111 studies (*N* = 5,046) reported 432 means for the sub-scale usefulness, 430 means for the sub-scale satisfying, and 34 means of the entire *Simple Scale*. Means presented in figures were estimated by the authors using lines in MS PowerPoint. Only 261 means of the scale and sub-scales (29%) were accompanied by corresponding standard deviations—a necessary condition to estimate standard errors for aggregating results across studies. Lastly, application contexts varied introducing critical heterogeneity into the data. Driver assistance systems were the most frequently researched topic. However, even within this study population applied technologies varied between haptic steering guidance, fatigue monitoring, congestion assistant, or forward collision warnings. These arguments—lack of standard deviations to estimate standard errors and heterogeneity of application context—inhibit any sensible calculation of aggregate scores of the *Simple Scale*.

The only tendency we could deduce from this database was generally larger usefulness than satisfying scores. Means for both sub-scales were reported in 424 instances across 97 studies (*N* = 4,095). In 318 of these cases (77%), the mean for usefulness was higher than the mean for satisfying across 15 different research topics.

## Discussion

This systematic literature review assessed applications of a “simple procedure for the assessment of acceptance of advanced transport telemetrics” [1]–a nine item semantic differential scale measuring acceptance with the two sub-scales usefulness and satisfying whose popularity is increasing and whose systematic evaluation has been pending. In sum, 128 publications with *N* = 6,058 participants provided results of the scale. In this section, we discuss findings about the scale followed by a reflection of how the scale was applied and how results were reported.

### Scale

Our most important finding questions the two-factor structure of the *Simple Scale*. Only two out of ten factor and principal component analyses were able to replicate both sub-scales. The combined sample size of these analyses (*N* = 869 in eight studies) outnumbered the original authors’ [1] own sample threefold producing more convincing results. Instead, the *Simple Scale* might produce a single acceptance score with high internal consistency (median Cronbach’s α = .90). Reported correlation coefficients between the two sub-scales were high (*r* ≥.55; four studies with *N* = 329 participants) suggesting a close relationship between usefulness and satisfying. This might explain why the two-factor structure was not replicated in the majority of factor and principal component analyses included in this review.

We thus recommend researchers who apply the scale to calculate explorative—or better confirmative—factor analyses with correlated factors and report their factor loadings together with model fit indices before using usefulness and satisfying scores. We refer to references [150–152] for more information on these procedures. Research on safety equipment in mobility or other emerging technologies with potential to disrupt markets relies on valid results. Objectivity (i.e., transparent and clear reporting) and reliability (i.e., checking test performances) are necessary to provide valid results and should thus be considered paramount in all fields of research.

As a second major finding, we identified the tendency that the *Simple Scale* produces higher means for usefulness than for satisfying in 77% of cases (97 studies with *N* = 4,095 participants). A first explanation for this finding is that indeed, the researched systems are more useful than satisfying. This might particularly be the case for systems that interfere with (driving) decisions of participants to increase safety. These systems might understandably be rated more useful than satisfying. However, the tendency was observed across 15 different research topics. Thus, an alternative second explanation points towards a possible method effect of the *Simple Scale* itself. Here, participants might be inclined to answer more affirmative to the five items for usefulness than to the four items for satisfying because of the items’ wording. A method effect would explain the finding of higher usefulness than satisfying scores across research topics. However, without the possibility to meta-analyse, both explanations seem probable and the result can only be seen as a tendency.

### Applications and reporting

Reporting of scale results was limited so that it was not possible to assess the scale using meta-analytic procedures. As examples, only 26% of reported means were accompanied by standard deviations, and only 22% of studies reported reliability coefficients. This was surprising since the original authors [1] themselves instructed researchers applying their scale to calculate Cronbach’s α as a measure of scale performance. We found that only half of the included studies (52%) received a quality score above.50 (scale range 0 to 1) using reporting of aims, sample characteristics, variable definitions, test performance, reproducibility, and missing data as indicators for study quality. These findings are worrying and need contextualising.

We identified that the *Simple Scale* is typically applied in papers predominantly concerned with technical aspects of a new system in transportation. Understandably, technical aspects (e.g., lateral offset, glace duration, or reaction times) are paramount for the systems’ performance and evaluation particularly in engineering and transportation research departments where most publications of the *Simple Scale* emerged. Ideally, subjective assessments using psychometric scales are applied and reported with as much rigour and conscientiousness as their objective technical counterparts. We thus urge researchers to critically reflect on their use of subjective measures and to report as extensively on the scales’ performance and results as journal guidelines allow. Only then is it possible to assess method effects and data structure using meta-analytical procedures.

## Supporting information

S1 File PRISMA 2009 checklist. (DOC)

S2 File Endnote library with search results and every step of the inclusion procedure. (7Z)

S1 Appendix Coding manual for data extraction. (DOCX)

S2 Appendix Coding manual for quality appraisal. (DOCX)

S1 Dataset Data extracted from 247 articles according to coding manual section A. (XLSX)

S2 Dataset Quality appraisal of 128 articles. (XLSX)

S3 Dataset Data extracted from 128 articles according to the entire coding manual. (XLSX)
