# Supplementary material for: Method-oriented systematic review on the simple scale for acceptance measurement in advanced transport telematics
Source: PLoS One. 2021 Mar 25;16(3):e0248107. doi: 10.1371/journal.pone.0248107 (PMC7993792; doi:10.1371/journal.pone.0248107)
Supplement: S1 Appendix — (DOCX) [file pone.0248107.s003.docx]

**S1 Appendix. Coding manual for data extraction.**

| Variable | Code | Description |
| --- | --- | --- |
| *Metadata* |  |  |
| Authors |  | All authors of the article |
| Publication year |  | Year of publication |
| Study title |  | Title of the text |
| Geographical setting |  | County of data collection; if not retrievable country of first author’s affiliation |
| Institutional link |  | Affiliations of each author |
| Article type | 0 | Not retrievable |
|  | 1 | Conference proceedings |
|  | 2 | Doctorate or graduate thesis |
|  | 3 | Report |
|  | 4 | Book or book chapter |
|  | 5 | Journal (not peer-reviewed) |
|  | 6 | Journal (peer-reviewed) |
| Journal name |  | For peer-review journals only |
| *Study design and procedure* |  |  |
| Domain of study | DAS | Driver assistance system |
|  | AUT | Automated driving |
|  | ISA | Intelligent speed adaptation |
|  | VSS | Vehicle safety systems |
|  | EV | Electric vehicles |
|  | ECO | Eco-driving |
|  | CARE | Care and nursing |
|  | CYC | Cycling |
|  | DSTR | Driver stress |
|  | DDTR | Driver distraction |
|  | HELI | Helicopters |
|  | EVHS | Extra-vehicular human safety |
|  | HMIF | Human-machine interface |
|  | HMIA | Human-machine interaction |
|  | IVS | In-vehicle signs |
|  | ORI | Operating room interactions |
| Study design | 0 | Not retrievable |
|  | 1 | Within-subject |
|  | 2 | Between-subject |
|  | 3 | Within- and between-subject |
| Research questions |  | Hypotheses or aim of the study |
| Sample size |  | Number of participants answering the VDL-scale |
| N men |  | Number of men participating |
| N women |  | Number of women participating |
| Mean age |  | Mean age of participants (calculated from categories) |
| Standard deviation age |  | Standard deviation of participants’ age (calculated from sub-samples) |
| Methods | 1 | (Driving) simulator |
|  | 2 | Field trial |
|  | 3 | Online Survey |
|  | 4 | Lab-test with mock-up equipment |
| Study outcomes |  | Results of the study |
| (Experimental) Conditions |  | Conditions of studies |
|  |  |  |

*(S1 Appendix continues)*

| S1 Appendix (continued) |  |  |  |
| --- | --- | --- | --- |
| Variable | Code | Description |  |
| *Simple Scale application* |  |  | |
| Simple Scale level |  | Reported level of the VDL-scale (e.g., 5-point, Likert or continuous) | |
| Simple Scale range |  | Reported range of the VDL scale (e.g., -2 to 2, 1 to 5, or -50 to 50) | |
| Presentation of scale results | 1 | Numbers |  |
|  | 2 | Bar chart |  |
|  | 3 | Two-dimensional diagram |  |
|  | 4 | Figure |  |
|  | 5 | Plain text |  |
|  | 6 | No reporting |  |
| Factor loadings USE |  | Loadings for each of the five items of the usefulness sub-scale |  |
| Factor loadings SAT |  | Loadings for each of the four items of the satisfying sub-scale |  |
| Median USE |  | Median of the usefulness sub-scale |  |
| Mean USE |  | Mean of the usefulness sub-scale |  |
| Standard deviation USE |  | Standard deviation of the usefulness sub-scale |  |
| Median SAT |  | Median of the satisfying sub-scale |  |
| Mean SAT |  | Mean of the satisfying sub-scale |  |
| Standard deviation SAT |  | Standard deviation of the satisfying sub-scale |  |
| Mean full scale |  | Mean of the entire VDL-scale |  |
| Standard deviation full scale |  | Standard deviation of the entire VDL-scale |  |
| Reliability coefficient USE |  | Cronbach’s alpha for usefulness sub-scale |  |
| Reliability coefficient SAT |  | Cronbach’s alpha for satisfying sub-scale |  |
| Reliability coefficient full scale |  | Cronbach’s alpha for the entire scale |  |
| *Relationships of VDL-scale* |  |  |  |
| Model variables |  | Constructs included in the conceptual model |  |
| Correlates of the VDL-scale |  | Correlations between sub-scales and with other constructs |  |
| Other statistics |  | Additional statistics used to answer research questions (e.g., t-tests, ANOVAs, or regressions) |  |
| *Miscellaneous aspects* |  |  |  |
| Translation of adjectives |  | Information about the use of translated versions of the VDL-scale |  |
| Miscellaneous |  | Other aspects about the VDL-scale (e.g., adaptation of the scale) |  |
| Team member |  | Initials of team members coding the studies |  |

*N* = 247 studies in section 1 (Metadata); *N* = 128 studies in all other sections.
