# Supplementary material for: Method-oriented systematic review on the simple scale for acceptance measurement in advanced transport telematics
Source: PLoS One. 2021 Mar 25;16(3):e0248107. doi: 10.1371/journal.pone.0248107 (PMC7993792; doi:10.1371/journal.pone.0248107)
Supplement: S2 Appendix — (DOCX) [file pone.0248107.s004.docx]

**S2 Appendix. Coding manual for quality appraisal.**

| **Variable** | **Verbal code** | **Numeric code** |
| --- | --- | --- |
| Specific aims of the study clearly stated? | Yes | 1 |
|  | No | 0 |
|  | Cannot tell | 0 |
|  | N/A | “N/A” |
| In studies in which ≥ two groups are compared, did investigators report how participants were chosen or allocated to groups? | Yes | 1 |
|  | Partially or somewhat | 0.5 |
|  | No | 0 |
|  | Cannot tell | 0 |
|  | N/A | “N/A” |
| Did the study define major variables? | Yes | 1 |
|  | Partially or somewhat | 0.5 |
|  | No | 0 |
|  | Cannot tell | 0 |
|  | N/A | “N/A” |
| Did investigators describe characteristics of enrolled sample, including important demographic factors? | Yes | 1 |
|  | Partially or somewhat | 0.5 |
|  | No | 0 |
|  | Cannot tell | 0 |
|  | N/A | “N/A” |
| When associations are reported, are effects of participant characteristics controlled for statistically or by design? | Yes | 1 |
|  | Partially or somewhat | 0.5 |
|  | No | 0 |
|  | Cannot tell | 0 |
|  | N/A | “N/A” |
| Did investigators document number of protocol violations, dropouts, subjects with incomplete data, etc.? | Yes | 1 |
|  | Partially or somewhat | 0.5 |
|  | No | 0 |
|  | Cannot tell | 0 |
|  | N/A | “N/A” |
| Was description of the procedure(s) clear enough for someone else to reproduce the study? | Yes | 1 |
|  | Partially or somewhat | 0.5 |
|  | No | 0 |
|  | Cannot tell | 0 |
|  | N/A | “N/A” |
| Are summary statistics for test's performance reported? (e.g., reliability or factor analyses) | Yes | 1 |
|  | No | 0 |
|  | Cannot tell | 0 |
|  | N/A | “N/A” |

*N* = 128 studies. Mean quality was calculated by adding all numeric codes divided by 8 minus the number of “N/A”’s.
